# Microbial Communities Associated with Primary and Metastatic Head and Neck Squamous Cell Carcinoma – A High *Fusobacterial* and Low *Streptococcal* Signature

**DOI:** 10.1038/s41598-017-09786-x

**Published:** 2017-08-30

**Authors:** Jae M. Shin, Ting Luo, Pachiyappan Kamarajan, J. Christopher Fenno, Alexander H. Rickard, Yvonne L. Kapila

**Affiliations:** 1 Department of Epidemiology, University of Michigan School of Public Health, Ann Arbor, MI USA; 2 Department of Biologic and Materials Sciences, University of Michigan School of Dentistry, Ann Arbor, MI USA; 3 Department of Periodontics and Oral Medicine, University of Michigan School of Dentistry, Ann Arbor, MI USA; 4 Department of Orofacial Sciences, The Division of Periodontology, University of California San Francisco, San Francisco, CA USA

## Abstract

Given the potential relationship between head and neck squamous cell carcinoma (HNSCC) and microbial dysbiosis, we profiled the microbiome within healthy normal and tumorous (primary and metastatic) human tissues from the oral cavity, larynx-pharynx, and lymph nodes using 16S rRNA sequencing. Alpha and beta diversity analyses revealed that normal tissues had the greatest richness in community diversity, while the metastatic populations were most closely related to one another. Compared to the normal, the microbiota associated with tumors supported altered abundances in the phyla *Fusobacteria*, *Firmicutes*, *Actinobacteria* and *Proteobacteria*. Most notably, the relative abundance of *Fusobacterium* increased whereas *Streptococcus* decreased in both primary and metastatic samples. Principal coordinate analysis indicated a separation and clustering of samples by tissue status. However, random forest analysis revealed that the microbial profiles alone were a poor predictor for primary and metastatic HNSCC samples. Here, we report that the microbial communities residing in the tumorous tissues are compositionally distinct compared to the normal adjacent tissues. However, likely due to the smaller sample size and sample-to-sample heterogeneity, our prediction models were not able to distinguish by sample types. This work provides a foundation for future studies aimed at understanding the role of the dysbiotic tissue microbiome in HNSCC.

## Introduction

With greater than 48,000 new cases each year in the United States and >500,000 cases diagnosed annually worldwide, head and neck squamous cell carcinoma (HNSCC) levies a major public health burden^1, 2^. Furthermore, the prognosis and the five-year survival rate of HNSCC have been constant for decades^3^. The known primary risk factors for HNSCC include tobacco and alcohol use, and infection by certain human papillomavirus (HPV) genotypes^4–6^. However, these risk factors alone have not been sufficient to explain the incidence and the mechanisms of tumorigenesis, and it is likely that other undescribed factors are playing important roles in HNSCC tumor development, progression and metastasis.

The human microbiome maintains a dynamic relationship with the human host^7^. For example, if the microbiome experiences an ecological imbalance, also known as dysbiosis, disease processes can emerge^8, 9^. Alternatively, changes in the human host, such as changes in the host adaptive immunity, can alter the associated microbiome^10^. Numerous studies have now reported that microbial dysbiosis is linked to cancer^11–14^. For example, imbalances in the gut microbiota promote altered host-microbial interactions that mediate colorectal cancer (CRC) tumorigenesis^15–17^. Genomic analysis of the microbiome of CRC patients have revealed a significant enrichment in *Fusobacterium* species with depletion in species from the phyla *Bacteroidetes* and *Firmicutes* relative to the normal healthy controls^15, 18^. Furthermore, Schmidt and colleagues reported that alterations in the oral microbiota were strongly associated with oral cancer^19^.

Close interactions between host cells and the microbiota will cause a variety of physiological responses in both the host and the host’s microbial inhabitants, including changes in individual microbes or in the collective microbial community. These interactions can be beneficial, neutral, or detrimental to the host. For example, bacterial communities within the gut maintain a mutually beneficial relationship with the human intestinal cells but in CRC, the increased abundance of certain bacteria (ie. *Fusobacterium nucleatum*) and their metabolic byproducts can potentiate and promote tumor growth by eliciting tumor promoting immune and host cell responses^17, 20, 21^. Accordingly, studies have suggested that local or distant cancer-associated microbiota can influence the cancer cells to exhibit cancer-specific inflammatory, immune and metabolic responses, or vice versa^22, 23^. In this study, we hypothesized that the local microbiota of HNSCC tissues have a distinct bacterial community profile compared to the healthy normal tissues. To understand whether bacterial organisms contribute to HNSCC development and progression or whether the abundance of bacterial organisms is altered in response to HNSCC development and progression, it is important to identify and analyze the associated microbial communities. If the tumor environment favors a specific microbial population or vice versa, further research is warranted to better understand these interactions in the development and progression of HNSCC.

## Materials and Methods

### Study Design and Human Subject Information

Normal and HNSCC human tissue specimens were obtained from ProteoGenex (ProteoGenex, USA). All clinical specimens were obtained with informed consents following standard protocols and with appropriate Institutional Review Board/Independent Ethics Committee (IRB/IEC) approval by the University of Michigan and ProteoGenex. Tissue samples were acquired based on availability. Tissues were snap-frozen in liquid nitrogen immediately following surgical removal and preserved at −80 °C until needed. In total, 72 tissue samples (normal, primary, metastatic) originating from the oral cavity, larynx, pharynx and lymph nodes of 34 HNSCC subjects (32 males and 2 females with an age range of 48–83 years and mean age of 59 ± 5.6 years) were used for this study. Among the collected tissue samples, i) matched normal adjacent, primary and metastatic HNSCC tissues were obtained from 14 subjects, ii) matched normal adjacent and primary HNSCC tissues were obtained from 10 subjects, and metastatic-only tissues were obtained from 10 subjects. We used each human subject as his/her own control (except the 10 metastatic non-matched samples). The subject specific information, including gender, age, tumor anatomic location, clinical diagnosis, TNM staging (extent of the tumor (T), extent of spread to the lymph nodes (N), the presence of distant metastasis (M) and tumor grade as established by histopathological evaluation), are included in Table 1.

**Table 1:** Human Subject Information.

Subject	Gender	Age	Location	TNM*	Grade	Sample type**
20	M	64	Larynx, Lymph node	T2N2bM0	G3	N, P, M
21	M	53	Larynx, Lymph node	T2N2bM0	G2	N, P, M
22	M	56	Larynx, Lymph node	T2N2M0	G3	N, P, M
23	M	54	Larynx, Lymph node	T3N2M0	G1	N, P, M
24	M	59	Oral cavity, Lymph node	T4aN1M0	G3	N, P, M
25	M	63	Laryngopharynx, Lymph node	T3N2M0	G3	N, P, M
26	M	71	Larynx, Lymph node	T4aN2bM0	G2	N, P, M
27	M	60	Pharynx, Lymph node	T3N2bM0	G3	N, P, M
28	M	53	Larynx, Lymph node	T4aN2cM0	G3	N, P, M
29	M	61	Larynx, Lymph node	T3N2cM0	G2	N, P, M
30	M	60	Larynx, Lymph node	T4aN2bM0	G3	N, P, M
31	M	57	Larynx, Lymph node	T3N2aM0	G2	N, P, M
32	M	54	Larynx, Lymph node	T4aN2cM0	G1	N, P, M
33	M	56	Larynx, Lymph node	T4aN2bM0	G3	N, P, M
2	M	62	Oral cavity	T3N0M0	G2	N, P, M
9	M	57	Oral cavity	T4N0M0	G1	N, P
10	F	83	Oral cavity	T3N0M0	G3	N, P
11	M	59	Larynx	T3N0M0	G2	N, P
12	M	50	Larynx	T3N0M0	G3	N, P
13	M	70	Larynx	T3N0M0	G2	N, P
14	M	59	Larynx	T3N0M0	G1	N, P
15	M	67	Larynx	T1N2bM0	G2	N, P
16	M	67	Larynx	T3N1M0	G2	N, P
18	M	60	Larynx	T3N0M0	G3	N, P
M1	M	51	Lymph node	T3N1M0	G2	M
M2	M	68	Lymph node	Recurrent	G2	M
M3	M	59	Lymph node	T3N1M0	G2	M
M4	M	48	Lymph node	T2N1M0	G1	M
M5	M	58	Lymph node	Recurrent	N/A	M
M6	M	58	Lymph node	Recurrent	N/A	M
M7	M	64	Lymph node	Recurrent	N/A	M
M8	M	62	Lymph node	Recurrent	N/A	M
M9	M	61	Lymph node	T3N2cM0	G2	M
M10	F	61	Lymph node	T2N2bM0	G1	M

All head and neck tumor samples examined in the study were clinically diagnosed and confirmed as squamous cell carcinoma.

The numbers in the ‘Subject’ column are for sample identification without any special meaning.

*TNM – TNM classification of malignant tumors is a cancer staging notation system; T describes the size of the original tumor and whether it has invaded nearby tissue; N describes the extent of lymph node involvement; M describes the presence of distant metastasis^24^.

**N – normal; P – primary tumor; M – metastatic tumor.

### RNA Extraction and cDNA Synthesis

Total RNA was isolated from the tissue samples using the RNeasy mini, RNA isolation kit (Qiagen, Germany) according to the manufacturer’s instructions. The cDNA was then synthesized using the high-capacity cDNA reverse transcription kit according to the manufacturer’s instructions (Applied Biosystems, USA).

### Microbiome Sequencing and Analysis

cDNA was normalized to 5 ng/μl per sample prior to running polymerase chain reactions (PCR). Targeted amplification and sequencing of the V4 variable region of the 16S rRNA gene was conducted in a single-step 30 cycle PCR using PCR primers 51/806^25^. The HotStarTaq Plus Master Mix Kit (Qiagen, Valentia, CA, USA) was used at the following conditions: 94 °C for 3 minutes, followed by 28 cycles (5 cycle used on PCR products) of 94 °C for 30 seconds, 53 °C for 40 seconds and 72 °C for 1 minute, after which a final elongation step at 72 °C for 5 minutes was performed. Genome sequencing was performed at MR DNA (www.mrdnalab.com, USA) on an Ion Torrent Personal Genome Machine (PGM) following the manufacturer’s guidelines.

Raw 16S data sequences were processed with QIIME 1.9.0. Samples with read counts less than 3000 preprocessing were excluded for microbiome analysis. Of 72 total samples collected, 71 samples had read counts over 3000. Sample M32 was excluded. Sequences with any ambiguous base calls, average Phred quality score below 25, max homopolymer length of >6, primer mismatch exceeding 0, or sequence length below 200 bp were discarded. All sequences that remained after filtering had primers, adaptors, and linker sequences truncated. Operational taxonomic units (OTUs) were clustered by 97% identity using the Uclust method. An open-reference OTU picking strategy was used where sequences that do not cluster against a reference database of sequences are clustered *de novo*. GreenGenes 13.8 was used as the 16S reference database. Sequences were aligned with GreenGenes-aligned sequences as template using PyNast. Taxonomy was assigned using the RDP Classifier in QIIME^26^. Singleton OTUs were filtered out as part of the default QIIME parameters. Additionally, OTUs constituting less than 0.05% of total reads were filtered out. The final OTU table was analyzed with QIIME and the Phyloseq package in R^27, 28^.

Downstream analytics included Shannon alpha diversity, community relative abundance, weighted UniFrac beta diversity, differential OTUs between HNSCC and healthy tissue samples. Outcomes were measured within the Phyloseq package and graphical output generated with R’s ggplot package. Additionally, beta diversity was visualized in 2-dimensional space with principal coordinate analysis using R’s built-in prcomp function. Log-transformed read counts of the OTU table was used as input for principal coordinate analysis.

### Random Forest Analysis

Random forest analysis (RFA) was used to predict normal or HNSCC status. The random forest regression modeling is a nonparametric approach, which accounts for the nonlinearities and interactions within the dataset to identify a subset of OTUs that are predictive of HNSCC. Another advantage of this approach is that cross-validation is built into the model generation procedure to limit the risks of over-fitting the model to the data^29^. Both primary and metastatic HNSCC tissues were considered different sample types that can be predicted by RFA. Ten RFA iterations were performed with random seeds 1–10 for each dataset where each iteration selected a random subset of 2/3 of the 41 samples to designate as the training dataset. The remaining 1/3 of the samples were designated as the testing dataset where random forest makes its best predictions on the sample types based on the training dataset.

### Statistical Analysis

Differences in Shannon alpha diversity as well as Euclidean distance for beta diversity between groups were tested using a non-parametric Kruskal-Wallis test. Differences in phyla abundances were evaluated using a non-parametric Mann-Whitney U test. Differential OTUs were detected using a Wald negative binomial test with the DEseq. 2 package in R. An α significance threshold of 0.05 used for the Kruskal-Wallis test and an α significance threshold of 0.01 was used for the Wald negative binomial test. A more conservative α threshold was selected for differential OTU tests to reduce the number of false positives that would be expected testing a large quantity of OTUs. To test whether microbial communities differ by HNSCC tissue type, an Adonis test that fits linear models to weighted UniFrac distance matrices was performed with R’s vegan package. Significance threshold indicating dissimilar communities was set at an α level of 0.05.

### Data Availability

The datasets generated during and/or analyzed during the current study are available from the corresponding author on reasonable request.

## Results

### Alpha and Beta diversity of Normal, Primary and Metastatic Tissue Samples

**Figure 1:**
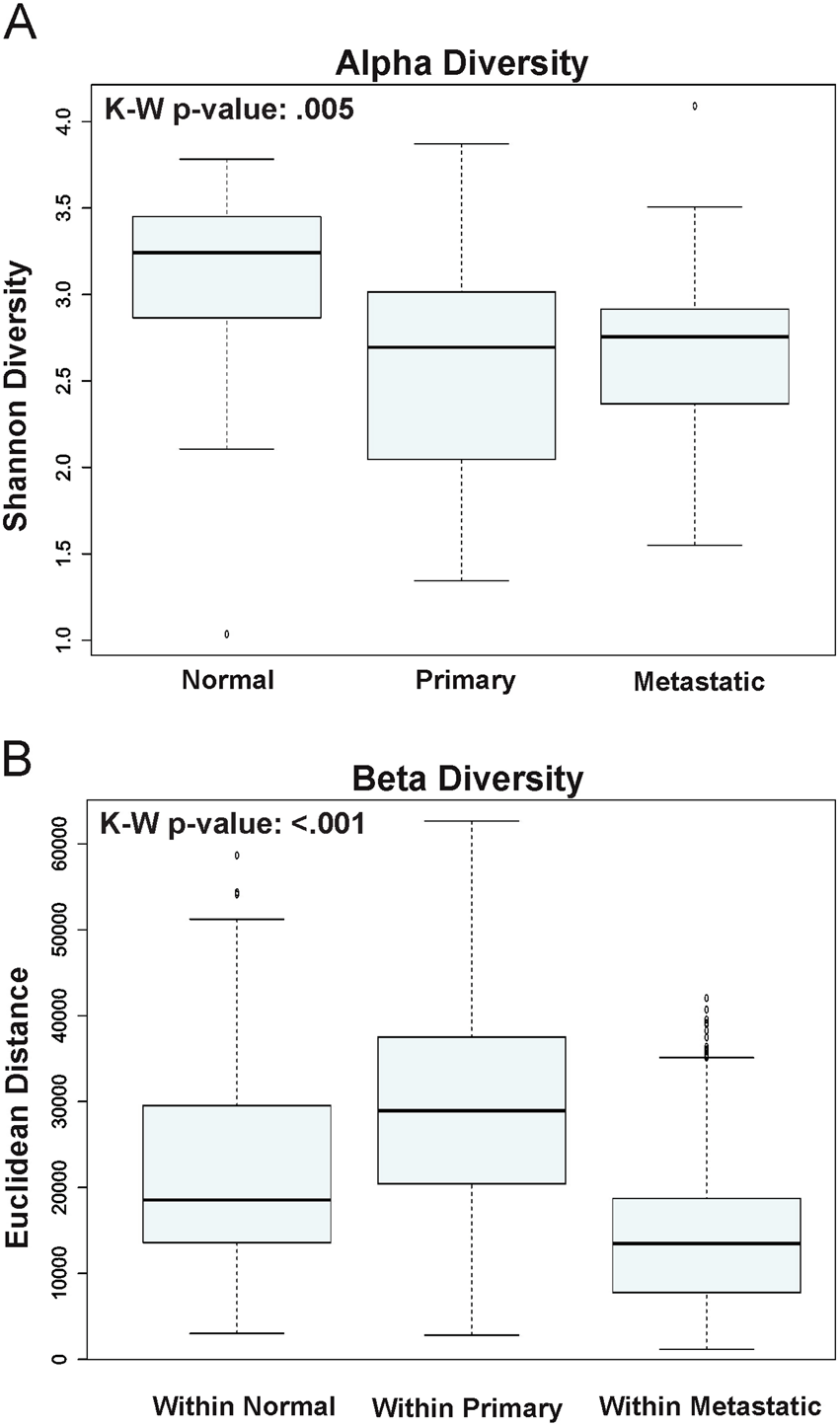
Alpha and Beta diversity of Normal, Primary and Metastatic Tissue Samples. (**A**) Alpha diversity based on the Shannon diversity index is shown for normal, primary and metastatic HNSCC tissue samples. (**B**) Beta diversity was measured by Euclidean distance for normal, primary and metastatic HNSCC tissue samples.

To compare the diversity captured from our samples, we conducted alpha and beta diversity analyses. Alpha diversity was calculated based on the Shannon diversity index, which measures the ecosystem biodiversity. The Shannon alpha diversity algorithm accounts for species richness and species evenness. Normal adjacent tissues had the greatest richness in community diversity compared to the primary and metastatic HNSCC tissue samples (Fig. 1A). The Kruskal-Wallis rank sum p-value was 0.005, indicating at least one pairwise comparison for Shannon alpha diversity was significant. The pairs driving significance were normal versus primary and normal versus metastatic (Fig. 1A).

For beta diversity, samples were clustered by each category level based on sample groups (normal, primary, metastatic) and each pairwise sample-to-sample dissimilarity was measured using Euclidean distance. Comparing across three sample types, the metastatic microbial taxa populations were more closely related to each other than to those in both the normal versus normal, and the primary versus primary HNSCC tissue samples (Fig. 1B). The p-values comparing within normal versus within primary, within normal versus within metastatic, and within primary versus within metastatic were all <0.001 (Fig. 1B).

### Phylum Distribution of the Normal, Primary and Metastatic HNSCC Tissue Samples

**Figure 2:**
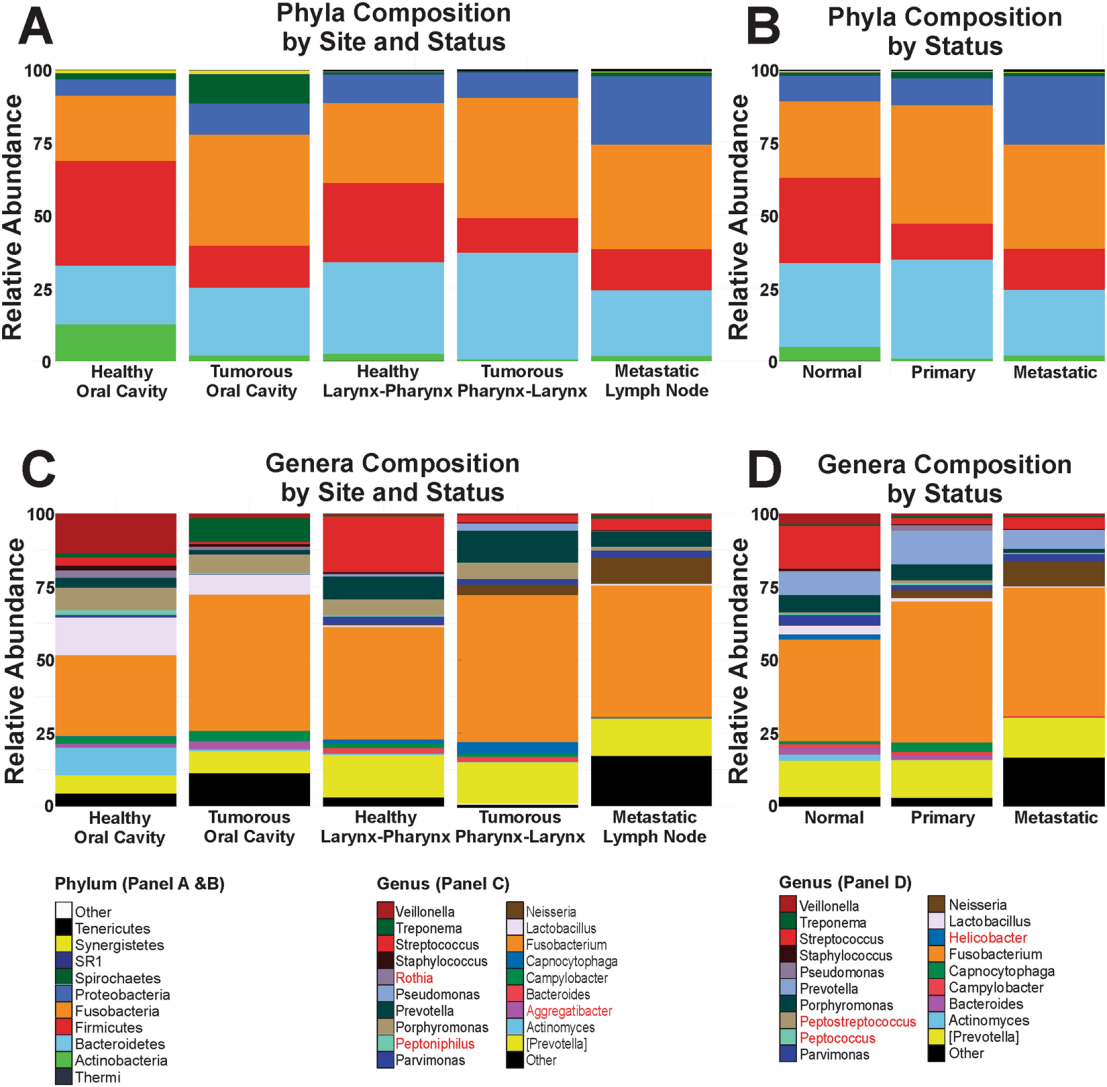
Phylum and Genus Distribution of the Normal, Primary and Metastatic HNSCC Tissue Samples. (**A**) The relative distribution of phyla based on anatomical locations is shown for normal, primary and metastatic HNSCC tissue samples. Matched samples were used for analysis. (**B**) The relative distribution of phyla based on tissue status. Matched and non-matched samples were pooled for analysis. (**C**) The relative distribution of genus based on anatomical locations is shown for normal, primary and metastatic HNSCC tissue samples. Matched samples were used for analysis. (**D**) The relative distribution of genus based on tissue status. Matched and non-matched samples were pooled for analysis. *Rothia*, *Peptoniphilus*, *Aggregatibacter* were detected in the top 20 genera by site and status not by status alone (Panel C), whereas, *Peptostreptococcus*, *Peptococcus* and *Helicobacter* were in the top 20 genera by status alone but not by site and status (Panel D).

Tissues were harvested from the oral cavity (lip and tongue), larynx and pharynx, and the mandibular lymph node (Table 1). To account for the differences in the microbiome profiles based on anatomic locations, community analyses were conducted by sample type and location (Fig. 2). Compared to the normal tissues from the oral cavity, primary HNSCC tissues showed increased abundance in *Bacteroidetes*, *Proteobacteria*, *Spirochaetes and Fusobacteria* (Fig. 2A). In addition, *Firmicutes* and *Actinobacteria* showed a marked decrease in abundance in the tumor tissues compared to the normal controls (Fig. 2A). Larynx and pharynx also exhibited prominent differences between the normal and the tumorous tissues, where *Fusobacteria* increased and *Firmicutes* decreased in relative abundance (Fig. 2A). As for the metastatic lymph node samples, the increased abundance in *Fusobacterium* species belonging to the phyla *Fusobacteria* and decreased abundance in *Streptococcus* species belonging to the phyla *Firmicutes* was consistent with that in tumor tissues from other locations. However, metastatic tissues selectively exhibited a higher prevalence of *Proteobacteria* (Fig. 2A). The community composition of the oral cavity (n = 8) and the larynx-pharynx (n = 40) region differed significantly, since *Firmicutes* and *Actinobacteria* flourished much more in the oral cavity (Fig. 2A). However, the power to detect statistical significance in the normal versus primary tumor group was hampered by the smaller sample size for each group. Overall, the microbiota of the tissues collected from the oral cavity exhibited greatest OTU richness (data not shown).

Based on the analysis conducted by tissue status, microbial species from *Firmicutes* and *Actinobacteria* were less abundant in both primary and metastatic HNSCC tissues compared to normal adjacent tissues (Fig. 2B). The relative abundance of *Firmicutes* was significantly lower in both primary and metastatic samples compared to the normal tissue samples (Table 2). The relative abundance of *Actinobacteria* was only significantly lower in primary HNSCC samples when compared to the normal samples (Table 2). The abundance of Fusobacterial populations was increased in both primary and metastatic tumor tissues compared to normal tissues (Fig. 2B); however, only the primary versus normal comparison was statistically significant (Table 2). Statistically significant differences in the abundance of Proteobacterial populations was present when comparing primary and metastatic tumor samples but not when comparing normal and primary tissue samples (Fig. 2B; Table 2). There was no significant difference in *Spirochaetes* abundance in each pairwise comparison by tissue type. The phyla *Tenericutes*, *Synergistetes*, *SR1* and *Thermi* represented less than 1% of the overall composition and their relative abundances were most disparate when comparing metastatic versus normal tissue samples (Table 2).

**Table 2:** The p-value matrix indicates the differential abundance of each phylum ﻿and genus between tissue samples by status and site.

Phylum	Normal Vs. Primary	Normal Vs. Metastatic	Primary Vs. Metastatic	Normal Vs. Primary (Oral Cavity)	Normal Vs. Primary (Larynx-Pharynx)
[Thermi]	0.0672	0.0338*	0.7808	N/A^a^	0.0640
Actinobacteria	0.0045*	0.1290	0.1134	0.2000	0.0094*
Bacteroidetes	0.9593	0.0459*	0.0298*	0.6857	0.8831
Firmicutes	0.0021*	0.0138*	0.2666	0.1143	0.0042*
Fusobacteria	0.0337*	0.1086	0.6351	0.4857	0.0634
Proteobacteria	0.3514	0.1345	0.0372*	0.3429	0.1738
Spirochaetes	0.4552	0.1334	0.3221	0.4857	0.2423
SR1	0.0655	0.0044*	0.4072	10.000	0.0385*
Synergistetes	0.1662	0.0260*	0.2746	0.8857	0.0923
Tenericutes	0.4258	0.0225*	0.1443	0.8857	0.4886
**Genus**					
[Prevotella]	0.6903	0.9580	0.7121	0.8857	0.5648
Actinomyces	0.0271	0.8398	0.0527	0.2000	**0.0375**
Aggregatibacter^b^	0.8930	0.6153	0.4999	0.3094	0.9675
Bacteroides	0.1904	**0.0017**	**0.0345**	0.8857	0.1478
Campylobacter	0.4803	**0.0077**	0.3922	0.0571	0.2012
Capnocytophaga	0.1472	**0.0102**	0.1474	0.8857	0.1022
Fusobacterium	0.0533	**0.0022**	0.2951	0.4857	0.0965
Helicobacter^c^	0.6609	0.8574	0.5445	0.4533	0.4989
Lactobacillus	**0.0401**	0.7738	0.1032	0.4857	**0.0498**
Neisseria	0.0926	0.0683	0.8726	0.3429	0.2034
Parvimonas	**0.0302**	0.4795	0.0535	0.6857	**0.0283**
Peptococcus^c^	0.8524	0.3385	0.2519	0.4857	0.9567
Peptoniphilus^b^	0.0832	0.8555	0.0700	0.8845	**0.0285**
Peptostreptococcus^c^	0.2199	0.5198	0.0866	0.4857	0.2977
Porphyromonas	0.1253	0.0508	0.6053	10.000	0.0634
Prevotella	0.9756	0.7438	0.8744	0.3429	0.8410
Pseudomonas	0.9753	0.4628	0.4125	0.6857	0.8181
Rothia^b^	**0.0003**	**0.0186**	0.3312	0.4857	**0.0002**
Staphylococcus	0.3514	0.1039	**0.0094**	0.4857	0.1572
Streptococcus	**0.0002**	0.1521	**0.0088**	0.2000	**0.0014**
Treponema	0.4188	0.2130	0.5160	0.4857	0.2211
Veillonella	**0.0182**	0.0719	10.000	0.4857	**0.0210**

The average phylum and genus abundance for each tissue sample compared to average phylum and genus abundance for each status (normal, primary and metastatic) and site is shown. Differences in abundance were examined using the Mann-Whitney U-test in R. Boldface* indicates a p-value < 0.05.

^a^Indicates no reads belonging to the phylum [Thermi] in both comparison groups. ^b^Top 20 genera when stratified by site and status, but not by status alone. ^c^Top 20 genera when stratified by status alone, but not by site and status.

### Genus Distribution of the Normal, Primary and Metastatic HNSCC Tissue Samples

Notable genus groups that increased in abundance in the oral cavity HNSCC tumor samples compared to the normal samples included *Fusobacterium* and *Treponema* (Fig. 2C). A marked decrease in *Streptococcus* and *Actinomyces* were observed in the HNSCC tissues (Fig. 2C). For larynx and pharynx samples, an increase in *Fusobacterium*, *Prevotella*, *Neisseria* and *Capnocytophaga* was observed, while a decrease in *Streptococcus* was observed (Fig. 2C). Furthermore, the genus *Lactobacillus*, *Parvimonas*, *Peptoniphilus*, *Rothia* and *Veillonella* were differentially abundant in the primary HNSCC samples collected from the larynx and pharynx compared to the normal samples (Table 2).

When the samples were pooled by status, compared to the normal to the primary HNSCC samples, the abundance of *Fusobacterium*, Prevotella, and *Capnocytophaga* increased whereas, *Streptococcus*, *Veillonella*, *Parvimonas*, *Lactobacillus* and *Rothia* significantly decreased (Fig. 2D, Table 2). In comparison to the normal metastatic samples, the abundance of *Fusobacterium*, *Neisseria* and other unknown genus groups increased, and *Streptococcus*, *Veillonella*, *Parvimonas* and *Lactobacillus* decreased (Fig. 2D; Table 2). In addition, other significantly altered genus included *Bacteroides*, *Campylobacter*, *Capnocytophaga* and *Rothia* (Table 2).

### Relative Abundance of Fusobacterial and Streptococcal Populations by Sample Types

**Figure 3:**
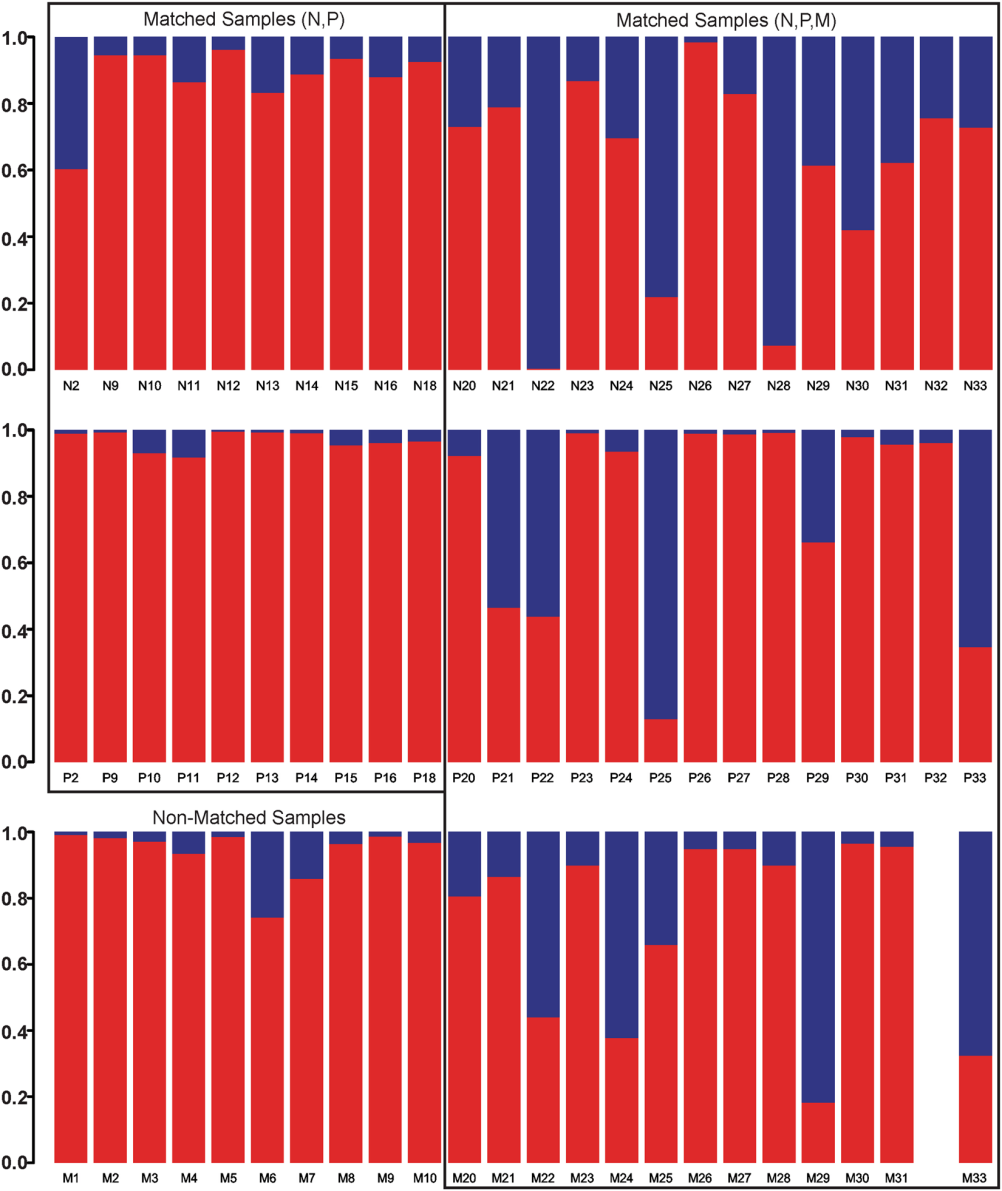
Relative Abundance of *Fusobacterium* and *Streptococcus* Population by Sample Types. The relative abundance of *Fusobacterium* and *Streptococcus* is shown for matched and non-matched samples. Matched groups are divided into two groups, one containing (N, P) and other (N, P, M) samples. The non-matched group contains only the metastatic samples. The red bars represent the abundance of *Fusobacterium* and the blue bars represent the abundance of *Streptococcus*.

The relative abundance of *Fusobacterium* and *Streptococcus* was compared by sample types. Matched samples were divided into two groups, 20 (N, P) samples and 41 (N, P, M) samples (Fig. 3). Ten non-matched samples were metastatic tissue samples (Fig. 3). In both matched groups, normal samples expressed greater abundance of *Streptococcus* species than primary HNSCC samples (Fig. 3). In both matched groups, the relative abundance of *Fusobacterium* species in primary HNSCC samples was much greater than the normal samples (Fig. 3). Matched metastatic samples exhibited more *Streptococcus* species than the primary samples, but were much less than the normal samples (Fig. 3). Both matched and non-matched metastatic samples exhibited lower abundance for both genera compared to the normal and primary samples (Fig. 3).

### Differential OTUs Detected between Normal, Primary and Metastatic HNSCC Tissue Samples

Wald negative binomial testing was performed to detect differentially abundant OTUs in 71/72 samples that contained more than 3,000 reads. In the primary versus normal tissue samples, there were 37 differentially abundant OTUs detected, with most belonging to the genera *Streptococcus* (24), *Fusobacterium* (4) and *Neisseria* (3). The direction of change was consistent across these 3 genera: Normal tissue samples had more *Streptococcus*, less *Fusobacterium*, and less *Neisseria*.

In the primary versus metastatic tissue samples, there were 60 differentially abundant OTUs. *Streptococcus* (26), *Actinomyces* (7), and *Fusobacterium* (5) genera constituted a significant portion of the differential OTUs. All of the *Streptococcus* and all of the *Actinomyces* OTUs were differentially abundant in the same direction: all were more abundant in the metastatic tissue samples. *Fusobacterium* OTUs (4/5) were less abundant in the metastatic samples. There was 1 *Fusobacterium* OTU that was more abundant in the metastatic tissue samples compared to primary tissue samples.

The comparison between normal versus metastatic tissue samples was the most disparate in terms of quantity of differential OTUs, which resulted in 104 OTUs. Some genera of note were *Streptococcus* (23 OTUs, and all of them were more abundant in normal samples), *Fusobacterium* (10 OTUs, with 8 that were more abundant in metastatic samples), and *Actinomyces* (6 OTUs, with all 6 more abundant in normal samples).

### Principal Coordinate Analysis (PCoA) Based on OTUs

**Figure 4:**
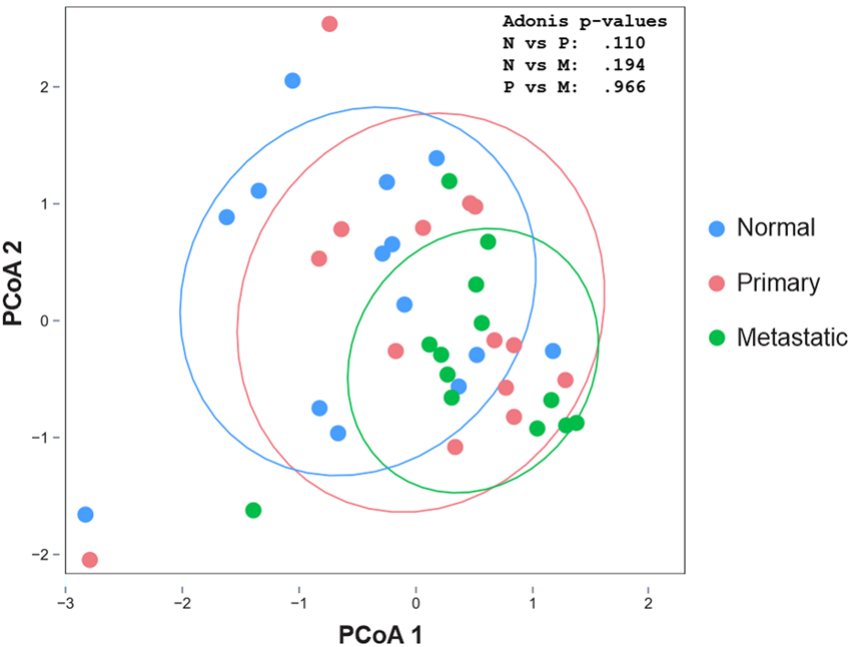
Distinguishing Normal and HNSCC Samples. Principal Coordinate Analysis (PCoA) was conducted based on the log-transformed read counts of the OTUs. Matched samples (Normal, Primary, Metastatic) were used for the PCoA.

Forty-one matched samples (normal, primary, metastatic) were used for the PCoA. A clustering pattern exhibited a left to right transition for a normal to primary then to a metastatic tissue status (Fig. 4). Across the sample population, the greatest clustering of communities was observed in the metastatic group, where the distances between samples were the smallest (Fig. 3). The ellipsoid boundaries of all 3 types of samples overlapped with one another. However, there was more substantial overlap in the microbial communities with most of the metastatic samples, such that the metastatic samples’ ellipsoid co-localized within the primary samples’ ellipsoid (Fig. 4). The separation between the normal versus primary (p = 0.11), normal versus metastatic (p = 0.194), and primary versus metastatic samples (p = 0.966) was not statistically significant as assessed by the Adonis test.

### Random Forest Regression Model to Predict HNSCC Using Microbial OTUs

The predictive accuracy of the random forest analysis was 39% (54/140). The majority of misclassifications were metastatic samples misclassified as primary tumor samples and primary tumor samples misclassified as metastatic samples (Table 3). When we categorized primary-metastatic samples as unhealthy, random forest analysis was able to better differentiate between the two groups. The predictive accuracy of correctly identifying an unhealthy (primary and metastatic) and normal sample increased to 76% and 59%, respectively. In aggregate, the predictive accuracy increased to 98/140 (70%) when the primary and metastatic tumors were combined under one umbrella as “tumor” tissue samples. Based on the RFA results, the outcomes coincided with the intersecting normal, primary and metastatic samples’ ellipsoids shown in the PCoA plot (Fig. 4).

**Table 3:** Random forest analysis (RFA) was conducted to predict the tissue status by OTUs.

48 Primary Samples	43 Metastatic Samples	49 Normal Samples	140 Total Samples
9 (19%) predicted correctly	16 (37%) predicted correctly	29 (59%) predicted correctly	54 (39%) predicted correctly
21(44%) misclassified as metastatic	23 (53%) misclassified as primary	14 (29%) misclassified as primary	86 (61%) misclassified
18 (37%) misclassified as normal	4 (10%) misclassified as normal	6 (12%) misclassified as metastatic	
**91 Unhealthy Samples**		**49 Normal Samples**	**140 Total Samples**
69 (76%) predicted correctly		29 (59%) predicted correctly	98 (70%) predicted correctly
22 (24%) misclassified as Normal		20 (41%) misclassified as unhealthy	42 (30%) misclassified

Matched samples (Normal, Primary, Metastatic) were examined to assess the accuracy of using microbial diversity to predict normal and HNSCC tissue conditions.

## Discussion

Bacteria in the human host often exist as compositionally diverse biofilm communities^30^. The environment created by the host influences the composition of the bacterial community, which is further shaped by other parameters, including temperature, oxygen tension, pH, substratum properties, nutrient availability, and exposure to cell and immune signaling^7, 31^. In this study, we evaluated the microbial communities of HNSCC tissues and their normal tissue counterparts. Analysis of alpha diversity revealed that normal tissues are significantly more diverse compared to the tumorous (primary and metastatic) tissues (Fig. 1A). Recently, Guerrero-preston and others reported that the saliva of HNSCC patients had significantly lower bacterial richness and diversity^32^. In this study, the beta diversity was greater in the primary HNSCC tissue samples and lower in the metastatic tissue samples (Fig. 1B). However, since the primary tumor tissues were harvested from different anatomic locations compared to the metastatic samples (lymph node), the greater beta diversity may partly reflect the differences in the microbiome profiles based on different biofilm habitats.

Based on our community analyses, the two major differences that were detected in these tissues were related to the abundance of members of the phyla *Fusobacteria* and *Firmicutes* (Fig. 2A and B). Compared to the normal tissue samples, an alteration of these two phyla was clearly observed within primary and metastatic tissue samples regardless of tissue status and location (Fig. 2A and B; Table 2). In both primary and metastatic HNSCC samples, *Fusobacteria* levels were increased, whereas *Firmicutes* and *Actinobacteria* were decreased compared to the normal samples (Fig. 2B; Table 2). In addition, a significant increase in *Proteobacteria* was observed in the metastatic samples (Fig. 2B). These data demonstrate that the host tumor microenvironment (TME) supports or is influenced by an altered microbial community.

Members of the genera *Fusobacterium* and *Streptococcus* are both highly abundant in the oral cavity^33^. In addition, *Fusobacterium* species are highly associated with periodontal disease^34^. *Fusobacterium nucleatum*, a Gram-negative anaerobe, is well known to coaggregate with both aerobic and obligate anaerobic bacterial species^35^. This strong coaggregative behavior elicited by *Fusobacterium* species is likely to provide additional benefits to the interacting species, beyond assisting with adherence and facilitating multi-species biofilm formation^36^. For example, *F*. *nucleatum* has the ability to adhere to and invade human gingival epithelial cells and help other bacteria to enter host cells by altering endothelial integrity^37, 38^. In solid tumors such as HNSCC, *Fusobacterium* species may play a role in providing protection for the tumor cells from the circulating immune cells. Gur and colleagues demonstrated that the presence of *F*. *nucleatum* inhibited tumor cell killing through inhibitory protein-receptor interactions with the immune cells^39^. The bacteria and tumor relationship is multifactorial and studies are starting to reveal clues about the specific role of bacteria in cancer. With the significant enrichment of a *Fusobacterial* population in primary HNSCC, these bacteria may be i) providing tumor cell immunity, ii) shaping the microbial community structure, and iii) providing benefits to the tumor cells by residing in the TME through bacteria-tumor cell interactions. Currently, studies are ongoing in our lab to further investigate the cellular mechanism of *Fusobacterium* species such as *F*. *nucleatum* in promoting HNSCC tumorigenesis.

Microbial community structure in a habitat is determined by the available nutrients, environmental conditions and the available colonizing species^36^. A hypoxic and pro-inflammatory TME may promote increases in abundance of certain bacterial populations, such as *Fusobacteria* and *Bacteroidetes*, while limiting the abundance of others like *Firmicutes* and *Actinobacteria* (Fig. 1A). In this study, an inverse relationship in the abundance of *Fusobacterium* and *Streptococcus* species was observed in the tumor tissues versus normal controls (Fig. 3). Schmidt and colleagues also demonstrated that this relationship was present in oral swab samples collected from oral cancer patients^19^. A significant reduction in the abundance of *Streptococcus* species and an increase in the abundance of *Fusobacterium* species was observed in oral cancer samples relative to the anatomically matched clinical normal samples^19^. In contrast, Gong and colleagues reported that *Streptococcus* dominated over *Fusobacterium* in the mucosa samples of laryngeal SCC patients^40^. Although these studies used different sample types (oral swabs, mucosal tissues, complex tissues) and different sequencing platforms (MiSeq, pyrosequencing, PGM), the inverse relationship relative to abundance between *Fusobacterium* and *Streptococcus* appears to remain robust.

In this study, we hypothesized that HNSCC tissues have distinct microbial communities compared to their normal healthy counterparts. If this community change occurs as the tissue transition from pre-malignant to malignant, these distinct microbial phenotypes might serve as risk indicators or predictors of disease status. For example, *Treponema denticola* is an oral *Spirochaete* that is normally found in low abundance in the oral cavity. In this study, the *Treponema* species were selectively increased in the primary tumor samples of the oral cavity (Fig. 2C). Frequent and preferential abundance of *T*. *denticola* has been associated with periodontal disease and esophageal tumor tissues^41, 42^. The oral treponemes are known to be resistant to host antimicrobial peptides (ie. human β-defensins), which can enhance the initial adhesion of other bacterial species to form the multi-species biofilm structures^43^. In addition, *Treponema* species are capable of inducing destruction of the host basement membrane structures through their innate proteases, which can further contribute to the tumor development and progression^44^.

Over the years, identification of strong risk factors, such as tobacco and alcohol use, and HPV infection, has proven to be useful indicators for HNSCC. Smoking can alter the bacterial acquisition and colonization of oral biofilms, and alter the composition of bacterial communities in saliva and biofilms in the subgingival pockets^45, 46^. In addition, Thomas and colleagues reported that bacterial richness was significantly reduced as a consequence of tobacco or alcohol use^47^. Recently, strong evidence has pointed to microbial dysbiosis as a causative or contributing factor to different types of cancer^12^. Alternatively, this dysbiosis may be the result of tumor development and progression. According to our PCoA plot, although not statistically significant, a microbial transition from a healthy to ‘HNSCC’ status can be seen with ellipsoids moving from a left to right direction along the PCoA 1 axis (Fig. 4). The primary and metastatic HNSCC samples pooled together with a tighter clustering pattern compared to the normal samples, and the greatest clustering was noted within the metastatic samples (Fig. 4). Further research is warranted to more closely examine the potential relationship between microbial shifts and HNSCC, and the specific role of microbial dysbiosis in HNSCC.

Recently, in a colon cancer mouse model system, RFA was successfully used to predict the behaviors of colon tumors. Specifically, Zackular and others predicted the final number of tumors based on the changes that occurred in the composition of the gut microbiota^48^. In this study, we utilized RFA to determine if we can predict the HNSCC (primary or metastatic) outcome by using the tissue microbiome data. Our results demonstrated that the accuracy was low in predicting primary and metastatic samples (19%, 37%), but greatly improved when we grouped the primary and metastatic samples into a single group as ‘unhealthy’ (76%; Table 2). The use of differential OTUs to predict the HNSCC outcome might not be sufficient, since the majority of the OTUs were shared between the normal and the HNSCC tissue types. In addition, unlike the Zackular study that generated predictions from a controlled animal model scenario, the current analysis was applied to human samples, which exhibit a greater level of heterogeneity, and therefore likely explain the lower predictive accuracy. However, it has been demonstrated that differential OTUs can successfully discriminate HNSCC tumor from control samples^32^.

The ideal clinical approach to improve the poor prognosis of HNSCC is through prevention and early detection and treatment. HNSCC is a complex multifactorial disease that is often only detected at advanced stages^3^. Hence, understanding the changes that occur in the microbiome associated with HNSCC tumors may provide a foundation for discovering new risk factors for early detection and diagnosis. In addition, a variety of omics biomarkers may be useful as early diagnostic tools for HNSCC^49^. In this study, we report on the alterations in microbial communities observed in primary and metastatic HNSCC tissues. Limitations of this study were i) relatively small sample size, ii) limited subject diversity and patient health history (specifically for tobacco usage, alcohol consumption and HPV status), and iii) heterogenic nature of microbial community based on tissue geography. In this study, we present findings that can serve as a key baseline data for future validation studies. If the altered microbiome is an important risk factor for HNSCC, it will be critical to understand its contributions along with those of other known risk factors. Although it is unclear whether the changes in the microbial composition cause or promote HNSCC or are the result of changes in the cellular activity of cancer cells, more comprehensive analyses involving tissue transcriptomics, proteomics, metabolomics and the microbiome will help better understand the role of host-microbial interactions in cancer.
